# Hybrid Lentivirus-phiC31-int-NLS Vector Allows Site-Specific Recombination in Murine and Human Cells but Induces DNA Damage

**DOI:** 10.1371/journal.pone.0099649

**Published:** 2014-06-23

**Authors:** Nicolas Grandchamp, Dorothée Altémir, Stéphanie Philippe, Suzanna Ursulet, Héloïse Pilet, Marie-Claude Serre, Aude Lenain, Che Serguera, Jacques Mallet, Chamsy Sarkis

**Affiliations:** 1 Unit of Biotechnology and Biotherapy, Centre de recherche de l'Institut du Cerveau et de la Moelle Epinière, Pierre-and-Marie-Curie University/Institut National de la Santé et de la Recherche Médicale, Paris, France; 2 NewVectys, Villebon-sur-Yvette, France; 3 Biosource, Paris, France; 4 Laboratoire de Virologie Moléculaire et Structurale, Gif-sur-Yvette, France; 5 Commissariat à l'Energie Atomique, Laboratoire de Radiobiologie et Oncologie, Fontenay-aux-Roses, France; 6 Molecular Imaging Research Center - Modélisation des biothérapies, Fontenay-aux-Roses, France; George Mason University, United States of America

## Abstract

Gene transfer allows transient or permanent genetic modifications of cells for experimental or therapeutic purposes. Gene delivery by HIV-derived lentiviral vector (LV) is highly effective but the risk of insertional mutagenesis is important and the random/uncontrollable integration of the DNA vector can deregulate the cell transcriptional activity. Non Integrative Lentiviral Vectors (NILVs) solve this issue in non-dividing cells, but they do not allow long term expression in dividing cells. In this context, obtaining stable expression while avoiding the problems inherent to unpredictable DNA vector integration requires the ability to control the integration site. One possibility is to use the integrase of phage phiC31 (phiC31-int) which catalyzes efficient site-specific recombination between the *attP* site in the phage genome and the chromosomal *attB* site of its *Streptomyces* host. Previous studies showed that phiC31-int is active in many eukaryotic cells, such as murine or human cells, and directs the integration of a DNA substrate into pseudo *attP* sites (p*attP*) which are homologous to the native *attP* site. In this study, we combined the efficiency of NILV for gene delivery and the specificity of phiC31-int for DNA substrate integration to engineer a hybrid tool for gene transfer with the aim of allowing long term expression in dividing and non-dividing cells preventing genotoxicity. We demonstrated the feasibility to target NILV integration in human and murine p*attP* sites with a dual NILV vectors system: one which delivers phiC31-int, the other which constitute the substrate containing an *attB* site in its DNA sequence. These promising results are however alleviated by the occurrence of significant DNA damages. Further improvements are thus required to prevent chromosomal rearrangements for a therapeutic use of the system. However, its use as a tool for experimental applications such as transgenesis is already applicable.

## Background

Gene transfer technologies are essential for genetics studies and gene therapies. However, major challenges remain to be addressed. A major issue is the lack of control over the site of DNA integration in the host genome which leads to unpredictable gene expression level and potentially undesirable mutagenesis of important cellular genes [1]. Recent strategies to tackle this challenge are relying on the use of genome editing tools such as ZFNs [2]–[7], TALENs [8]–[13] or more recently CRISPR-Cas system [14]–[17]. However, the vectorization of these tools into viral vectors to optimize their use *ex vivo* or *in vivo* raises several problems. Indeed, ZFNs function as dimers and generally require cotransduction of three vectors (one for each dimer and one for the recombinating substrate) [18]–[20]. Moreover ZFNs may induce cellular toxicity due to off target activity [21]–[23]. TALENs have an important size with repeat domains hampering their vectorization [24]. CRISPR-Cas is a very recent tool and its vectorization has not yet been described. One may however expect its vectorization into viral vectors will be challenging as the system is based on the concomitant use of a chimeric DNA displaying hairpin structures [25] and of Caspase 9 which induces apoptosis when over-expressed [26]. These features will undoubtedly represent challenges for the vectorization of CRISPR-Cas into viral vectors for targeted integration.

**Figure 1:**
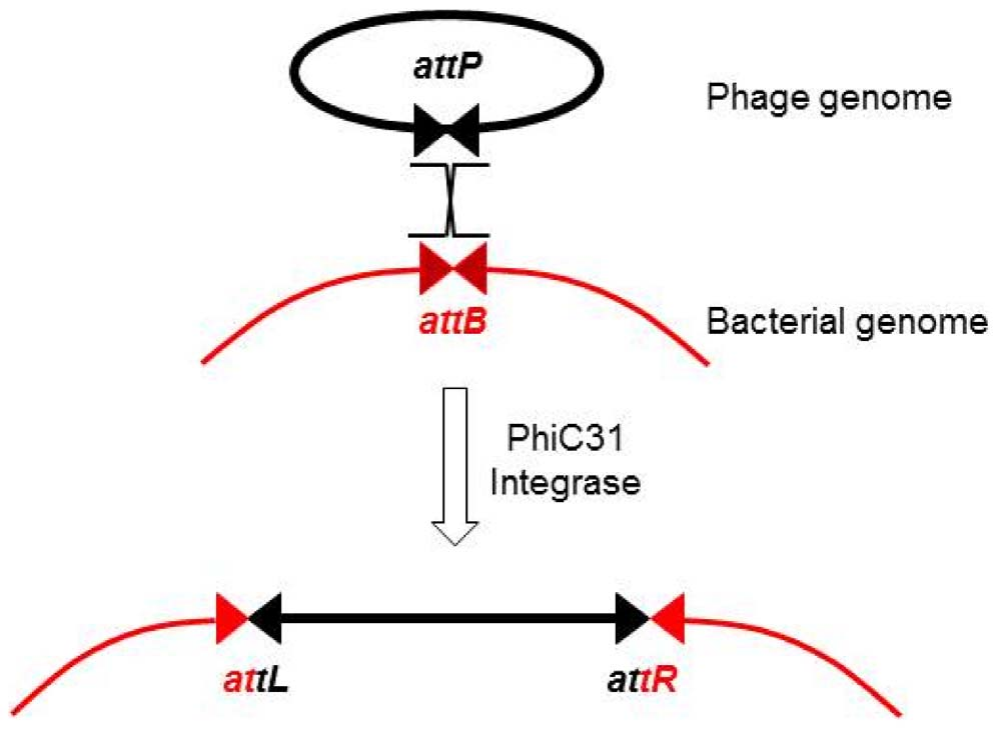
Scheme of phiC31-int mediated recombination in bacterial host. PhiC31 integrase performs precise recombination between an *attB* site located in the *Streptomyces* genome and an *attP* site located on the phiC31 phage genome. The outcome is integration of the phage into the host genome.

Site-specific recombinases such as Cre [27]–[34] or FLP [35]–[38] of the tyrosine recombinases family are other genome editing tools more easily vectorizable and widely used for the purpose of site specific integration. However, the use of these recombinases is limited by the absence of endogenous recognition site in mammalian cells and by the bidirectionality of the recombination reaction they mediate. Within the superfamily of site-specific recombinases, phage integrases catalyse unidirectional recombination events [39]. Among these the PhiC31 phage integrase (phiC31-int), of the large serine recombinases family, is the most commonly used site-specific integrase for gene transfer purposes [40], [41]. In its natural context, phiC31-int mediates efficient recombination between the phage attachment site (*attP*) and the bacterial attachment site (*attB*). The recombination of these two sites results in the unidirectional and site-specific integration of the phage genome into the bacterial chromosome (reviewed in [39]) leading to an integrated phage genome flanked by the recombinant *attL* (left) and *attR* (right) sites (Figure 1). When used for gene transfer into eukaryotic cells, phiC31-int can catalyse integration of a plasmid containing an *attB* sequence into endogenous pseudo *attP* sites (p*attP*) displaying a high degree of homology with the wild type *attP* site [42]. Hence, associated with transfection techniques, phiC31-int has been successfully exploited to stably modify the genome into particular genomic sites of many types of eukaryotic cells *in vitro* [43]–[50] for transgenesis [48], [51]–[55] and gene therapy applications [56]–[65]. The use of phiC31-int presents several advantages. First, the recombinase can be used to generate conservative recombination between *attB* and pseudo *attP* sites [42]. Second, Chalberg et al demonstrated that the majority of phiC31-int mediated recombination events in the human genome occur in intergenic regions [66]. However, in most of these studies the vectorization of phiC31-int relied on cotransfection (or nucleofection) of plasmids for both the delivery of phiC31-int and of the transgene, thus limiting this technology to *in vitro* or *ex vivo* applications.

A strategy to increase the efficiency of DNA delivery *in vivo* is to use viral-derived vectors. As the expression of the genome editing tool must be transitory to avoid genotoxicity, it could therefore be delivered by a transient viral vector [67]. Even though phiC31-int has already been delivered by an adenoviral vector system [68], [69] the use of such vectors is limited by their cell toxicity and immunogenicity [70]–[72]. In contrast, lentiviral vectors (LV) have the advantage to be non-immunogenic [73], [74] and offers the possibility to be pseudotyped by different envelops, allowing a high degree of flexibility regarding the tropism of the particles and the type of cell(s) they transduce (for review see Cronin J. et al [75]). Most importantly, it was shown that LV integrase can be modified to obtain non integrating lentiviral vectors (NILVs) [76]–[78], which act as episomal vectors. Hence, NILVs are vectors of choice to deliver genome editing tools and have been successfully used to deliver transposases [79], [80], FLP [81] or ZFNs [82], [83]. However, the use of NILVs has never been described for the vectorization of a serine recombinase.

In the present study, we combine the unidirectional site-specific recombination capability of phiC31-int with the efficiency of NILVs for gene transfer. For targeted integration,two different NILVs, one delivering the DNA sequence to be integrated and containing the natural *attB* site, the other expressing the phiC31-int are used. Through a step by step approach, we demonstrate for the first time that phiC31-int can be vectorized in NILV and we provide clues to further improve the system. However, analysis of integration events reveals that significant DNA damages can result from phiC31-int mediated recombination. In conclusion, the vectorization of serine recombinases in NILVs is feasible and constitutes a promising tool for basic research; however, one should remain cautious about the chromosomal aberrations that can be induced by these recombinases, particularly for clinical uses.

## Results and Discussion

### NILV genomes can be used as a substrate for site-specific integration mediated by phiC31-int into human genome

**Figure 2:**
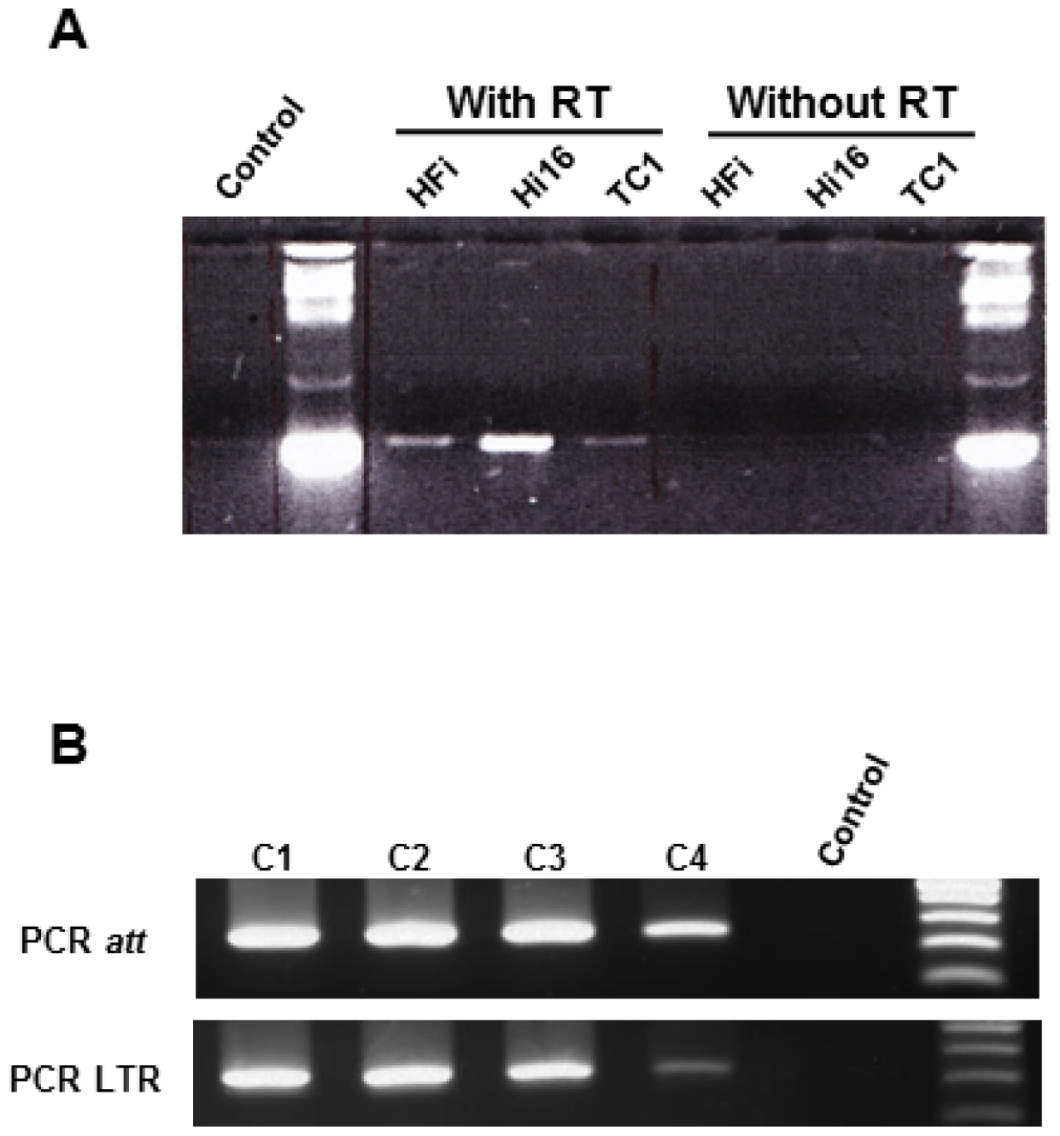
Analysis of cell lines which constitutively expressed phiC31-int. A) PhiC31 RT-PCR on three different cell lines. HFi and Hi16 are derived from Hela cell line and TC1 from NIH-3T3 cell line. Control condition lane lacks RNA. B) PCR which detects LTR junctions or intact *attB* sites after transduction with a NILV *attB*-CMV-Neo.

We first assessed the ability of a NILV DNA genome to be used as a substrate for phiC31-int. We therefore generated a Hela cell line constitutively expressing phiC31-int thanks to an integrative lentiviral vector expressing the phiC31-int under the control of the CMV promoter (LV CMV-phiC31-int) to. Hela cells were transduced and clonal populations were isolated and analyzed by RT-PCR to estimate the phiC31-int expression level (Figure 2A). The clone Hi16 was selected for its robust constitutive expression of phiC31-int.

**Figure 3:**
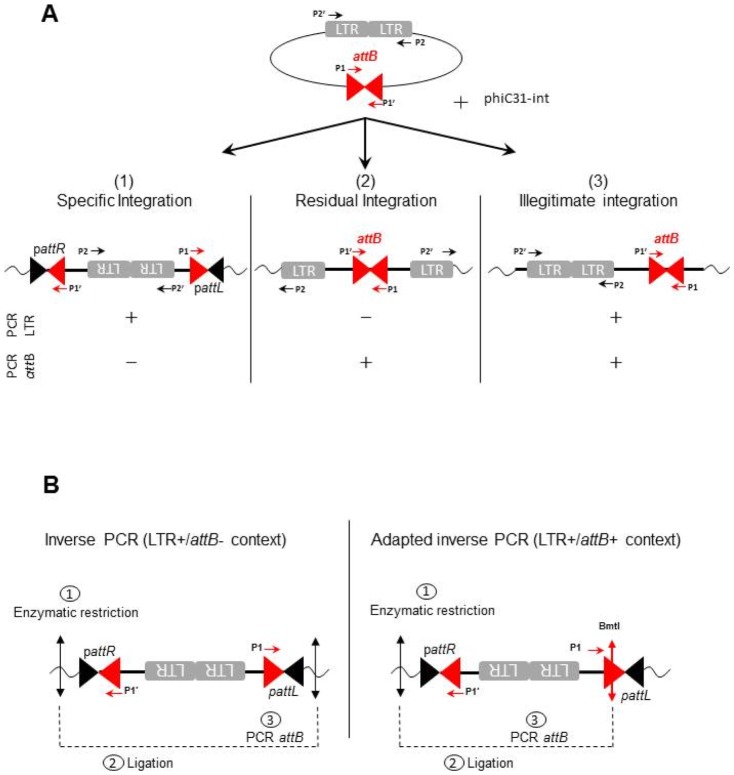
Analysis strategies to detect the specific integrations mediated by phiC31-int. A) Illustration of the three mechanisms of the phiC31-int mediated integration of a NILV containing an *attB* sequence. According to the type of integration, the PCR results in three different profiles: - PCRs LTR+/*attB*− : integration type (1), specific integration. - PCRs LTR−/*attB*+: integration type (2), residual integration. - PCRs LTR+/*attB*+: integration type (3), illegitimate integration. P1/P1′ are the primers used for *attB* PCR and P2/P2′ are the primers used for LTR PCR. B) Schematic representations of the inverse PCR and the adapted inverse PCR strategies used to characterize phiC31-int integration sites.

The ability of the constitutively expressed phiC31-int to mediate recombination between a NILV bearing an *attB* site with genomic p*attP* sites was then tested. Hi16 cells were transduced with a non-integrative lentiviral vector expressing the Neomycine (Neo) resistance gene under the control of the CMV promoter and containing an *attB* site (NILV a*ttB*-CMV-Neo). After two weeks of G418 selection we obtained four cell clones which genomes were analyzed. Theoretically Neo integration is expected to arise from 3 distinct mechanisms: (i) phiC31-int-**specific** integration, (ii) **residual** integration mediated by a residual activity of the mutant HIV integrase (review about this field [84]) or (iii) **illegitimate** integration due to recombination of the episomal DNA molecule with the cellular genome by host cell mechanisms (Figure 3A). If the analysis is realized with clonal populations, these three different mechanisms should be discriminated by performing 2 PCR assays, one amplifying the LTR and the other amplifying the a*ttB* site. A positive LTR PCR reveals the presence of a LTR-LTR junction, indicating that the integration has occurred through a LTR-independent mechanism, either involving *attB* site-specific recombination or illegitimate recombination. In contrast, a positive a*ttB* PCR reveals that the a*ttB* site is intact, indicating an a*ttB*-independent integration, either involving LTR dependent (residual) integration or illegitimate recombination. In summary, cells are analyzed without knowing whether their genome contains one or several integration of NILV. A positive result for LTR PCR only or for *attB* PCR only allows to identify the mechanism of integration without ambiguities (ie respectively phic31-int specific integration or residual integration). As the LTR PCR does not amplify the 1-LTR region generated when the linear genome circularized through homologous recombination of both LTR regions, recombination involving 1-LTR circles would always result in LTR− in the PCR test. In cells where a single integration of a 1-LTR circle event arose, attB+/LTR− or attB−/LTR− profiles may be detected, respectively when the recombination is independent or dependent on phiC31-int. In all our experiments, we never observed attB−/LTR− clones. On the contrary, if both PCR are positive integration could result either from illegitimate recombination or from double independent recombination events (ie: phiC31-int site specific recombination and HIV integrase mediated residual integration). We collected the four clones and checked integration pattern of NILV with PCR. The PCR analysis revealed that all clones were positive for both LTR and a*ttB* PCRs (Figure 2B). Thus this result does not allow to conclude about the nature of the integration events.

**Figure 4:**
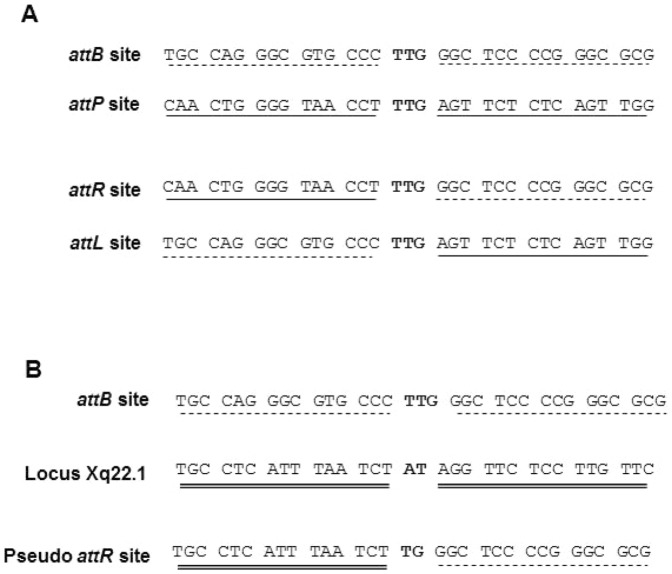
DNA sequence of *att* and p*attP* sites. A) Wild type *attP* and *attB* sites. After recombination two hybrids sites are formed: *attL* and *attR*. B) Recombination between *attB* site and the human locus Xq22.1 This recombination generates a p*attR* which has been isolated by inverse PCR. Xq22.1 had been described previously as a human p*attP* by MP Calos et al., who isolated the same p*attR*.

To clear up this ambiguity, genomic DNA of isolated clones was further analyzed by inverse PCR (iPCR) to isolate potential specific integration events. *attB* based primers (P1/P1′) were used to amplify junctions of recombination. To avoid the PCR background generated by the non-recombined *attB* site, an enzymatic cocktail including an enzyme which cuts only once in the vector sequence, in 5′ to the *attB* site (Figure 3B) was used. In this way, we were able to isolate pseudo *attR* junctions with iPCR, (but not the pseudo *attL* junction). Using this adapted strategy, a lentiviral genome integrated by phiC31-int at the human locus Xq22.1 was detected (Figure 4). Interestingly this locus had already been described as a preferential p*attP* site in the human genome [66]. Moreover, the core sequence is exactly in the same position, suggesting that the recombination event occurred very precisely at this locus.

Although NILV integration also occurred through other means than phiC31-int recombination, our results clearly demonstrate that a NILV is a suitable substrate for phiC31-int mediated recombination in human cells. We therefore investigated whether phiC31-int could function when vectorized in a NILV.

### NILVs allow adequate expression of phiC31-int to mediate recombination into a reporter system containing an a*ttP* site artificially introduced in human cells

**Figure 5:**
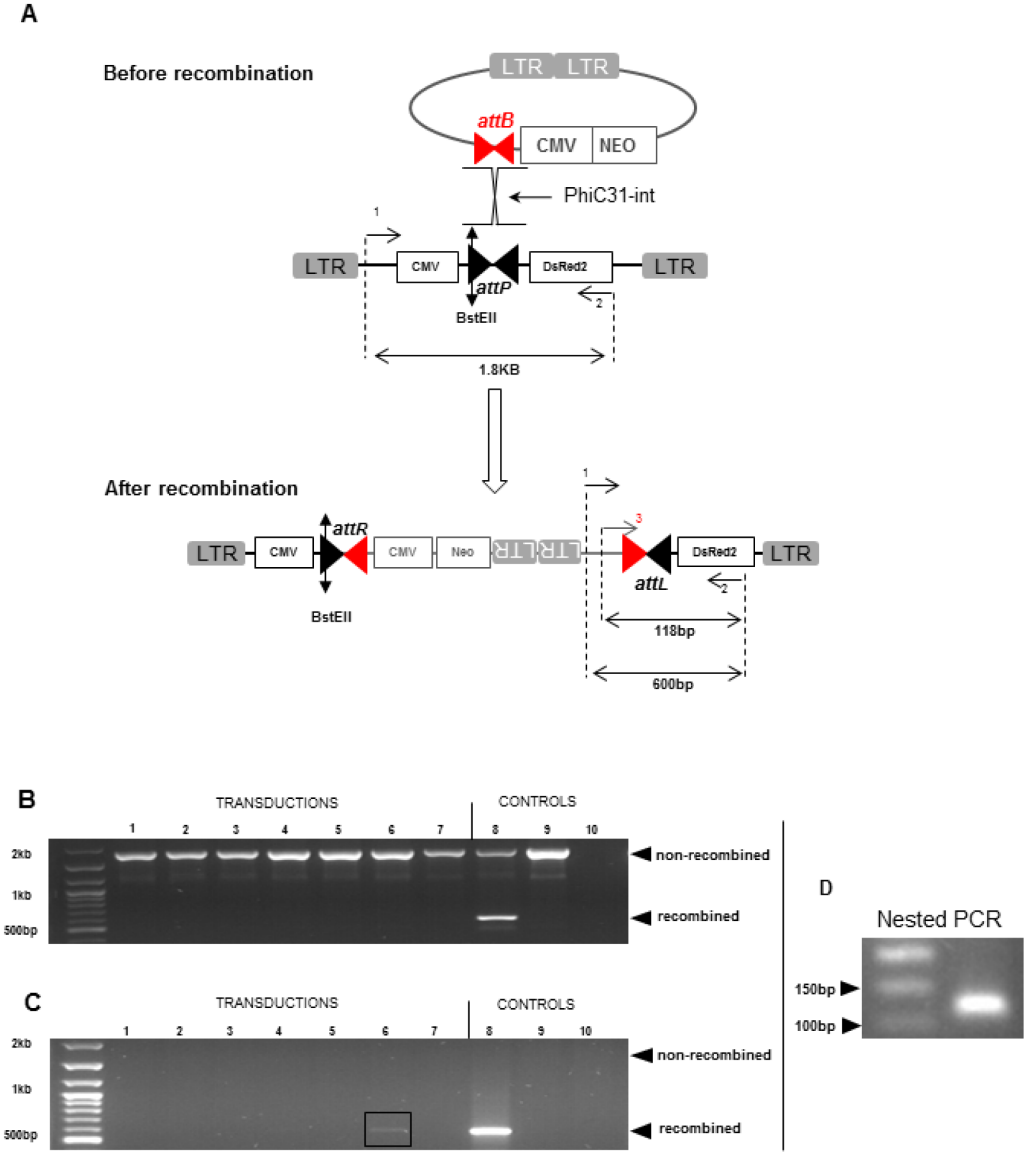
Detection of recombination mediated by phiC31-int between an *attB* site contained into a NILV and a genomic *attP* site. A) Scheme of the DsRed2 PCR before and after the enzymatic restriction treatment. B) PCR DsRed2 results without restriction enzyme treatment. Lanes 1 to 3: cotransduction with CMV-Neo and CMV-PhiC31 increasing vector input of 50–150–300 ng of p24. Lanes 4 to 6: cotransduction with *attB*-CMV-Neo and CMV-PhiC31 increasing vector input of 50–150–300 ng of p24. Lane 7: *attB*-CMV-Neo. Lane 8: positive control generated by triple-transfection (CMV-phiC31-int, *attB*-CMV-Neo and CMV-*attP*-DsRed2). Lane 9: negative control without vector. Lane 10: negative control of PCR. C) PCR DsRed2 results after restriction enzyme treatment. Lanes are similar to figure B. D) Nested PCR from the product isolated from lane 6 to confirm the specificity of PCR DsRed2 amplification.

The ability of a NILV to express phiC31-int and mediate recombination between another NILV genome carrying an *attB* site and a wild-type *attP* site artificially introduced in a human cell genome was further tested. First, a clonal Hela cell line containing the wild-type *attP* site inserted in its genome (HDsred line) was generated using an integrative LV (CMV-a*ttP*-DsRED2). HDsred cells were then transduced with two NILVs, one allowing expression of phiC31-int (NILV CMV-phiC31-int), and the other expressing Neo and containing the *attB* site (NILV *attB*-CMV-Neo). After cotransduction cells were grown with G418 to select integration events. The genomic DNA extracted from Neo resistant clones was analyzed by PCR to detect a*ttL* recombination junction (Figure 5A). Results showed only background signal generated by non-recombined *attP* site (figure 5B). To prevent this amplification, the genomic DNA was digested by a restriction enzyme that cuts both *attP* and *attR* sites but not *attL* site (Figure 5A) prior to PCR amplification. PCR results obtained after the enzymatic treatment revealed an a*ttL* junction in the population transduced with the highest dose of phiC31-int vector (Figure 5C). These results have been further confirmed by nested PCR (Figure 5D) and PCR product sequencing. Taken together, these results demonstrate that a NILV can deliver a functional phiC31-int capable to integrate an episomal lentiviral substrate containing an *attB* site into an *attP* site artificially introduced into the human genome.

Although the used PCR strategy does not allow to estimate the targeting efficiency of the *attP* site with the double vector system, the need of a nuclease digestion before PCR to reveal specific integration events suggests that the efficiency of the two NILVs system is low. As this assay involved the two natural recombination sites of PhiC31-int, further reduced efficiency would be expected when targeting endogenous p*attP* sites. Consequently, we next focused on the improvement of the two NILVs system efficiency.

### Modification of the phiC31-int sequence to improve the efficiency of the NILV phiC31-int to allow target integration in pseudosites a*ttP*

**Figure 6:**
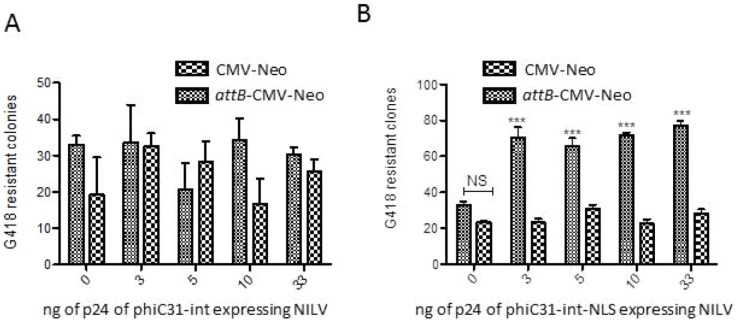
Effect of NLS sequence on phiC31-int activity in NILV context. A) Cotransduction of NILVs CMV-PhiC31 and CMV-Neo or *attB*-CMV-Neo. Four p24 doses of PhiC31 vector were used (D1: 3 ng, D2: 5 ng, D3: 10 ng, D4: 33 ng). B) Cotransduction of NILVs CMV-PhiC31-NLS and CMV-Neo or *attB*-CMV-Neo. Four p24 doses of PhiC31 vector were used (D1: 3 ng, D2: 5 ng, D3: 10 ng, D4: 33 ng). No significant differences are observed between sample with or without a*ttB* sequence in the vector pTRIP-CMV-Neo. Satistics: two ways ANOVA with Bonferroni posttest (Prism 5).

It was previously shown that C-terminal addition of a nuclear localization system (NLS) to phiC31-Int improves its efficiency in eukaryotic cells [85]. We therefore tested this improved integrase in NILV vectorization strategy. To compare the two versions of phiC31-int, Hela cells were cotransduced with the following NILVs vectors: CMV-Neo with or without *attB* site and CMV-phiC31-int with or without NLS. Cells were grown with G418 and the resistant clones were quantified. The results from experiments in which CMV-phiC31-int was cotransduced with either CMV-Neo or *attB*-CMV-Neo are presented in Figure 6A. The CMV-Neo and the *attB*-CMV-Neo conditions did not display significant differences, indicating that no significant PhiC31-int recombinase activity occurred. In contrast, when the cells are transduced with CMV-phiC31-int-NLS instead of CMV-phiC31-Int (Figure 6B) the presence of the NLS sequence induced significant differences between the CMV-Neo and the *attB*-CMV-Neo conditions. These results show that addition of a C-terminal NLS to phiC31-int significantly increased its recombination efficiency with p*attP* sites. Indeed the phiC31-int-NLS mediated integration was 2 to 2.5 fold above the background level produced by NILV residual integration (Figure 6B).

The use of the phiC31-int-NLS vectorized in a NILV allows to significantly increase the efficiency of recombination. We therefore further tested this hybrid lentivirus phiC31-int-NLS vector to target genomic p*attP* site into murine and human cells.

### The two NILVs system allows site specific recombination in murine and human cells but induced aberrant chromosomal rearrangements

To target pa*ttP* site in the murine and human genome with the two vectors system, Hela cells and NIH-3T3 cells were cotransduced with the NILV *attB*-CMV-Neo and the hybrid lentivirus phiC31-int-NLS vector. After two weeks of selection, Neo resistant cells were collected and several clonal populations were isolated to facilitate interpretation of PCR analyses. The clones were analyzed with the LTR and a*ttB* PCRs assay (Figure 3A) to determine the proportion of specific integration events compared to NILV residual integration and illegitimate recombination events. We categorized clones in the 3 following groups: LTR+/a*ttB*− clones (group I, ie phiC31-int-NLS integration), LTR−/*attB*+ clones (group II, ie residual integration) and LTR+/*attB*+ clones (group III, ie illegitimate integration or mixed integration profile). The results are presented in Table 1.

**Table 1:** Repartition in 3 groups of human and murine clones according to the *attB* and LTR PCR results.

		Group I (LTR+) *Specific integration*	Group II (attB+) *Residual integration*	Group III (LTR+/attB+) *Unknow integration*
**Mouse**				
	Clone number	8	78	22
	%	7,5%	72,2%	20,3%
**Human**				
	Clone number	13	15	0
	%	46,4%	53,6%	0,0%

We obtained 108 clones for murine cells and 28 for human cells. 7.5% of murine clones are in group I and 20.3% in group III (Table 1A). Consequently the integration mediated by the hybrid lentivirus phiC31-int-NLS vector is comprised between 7.5% and 27.8%. As no human clone corresponded to group III, the proportion of the hybrid lentivirus phiC31-int-NLS vector specific integration corresponds to the proportion of group I, ie 46.4% (Table 1B).

To determine which integration sites were targeted and confirm the type of integration of clonal groups I and III, we performed an analysis by iPCR as previously described (Figure 3B). The sequencing of iPCR products demonstrates that all human and murine clones tested contain in their genome an integrated vector with a recombinant pattern at the *attB* site. Therefore, the two vectors system that we developed allows targeting p*attP* sites in the murine and human genomes (Table 2).

**Table 2:** Mapping and description of pa*ttP* sites isolated by iPCR on human and murine cell lines.

			Deletions		Genomic location		If intronic	If intergenic, flanking gene names			
	Chromosome	Number of junction isolated	The half of *att* site present into the vector	Chromosome	Pseudo site	Context	gene name	5′side gene	Distance (kb)	3′side gene	Distance (kb)
**Mouse**											
**Groupe I**	**1**	2	**p** ***attR*** **: 2 bp** **p** ***attL*** **: 42 bp**	ND	1E4	repeat sequence					
	**2**	2	**p** ***attR*** **: No** **p** ***attL*** **: 13 bp**	ND	2H3-H4	repeat sequence					
	**7**	2 (only p*att*R exploitable)	**p** ***attR*** **: 4 bp**	ND	7F2108099955	Exonic	MOR204-16				
**Groupe III**	**1**	1	**p** ***attR*** **: 8 bp**	ND	1H1159228123	Intergenic		PAPP-A2	277	AI316802	49
	**5**	1	**p** ***attR*** **: 16 bp**	ND	5B129812070	Exonic	4632413E21Rik				
	**5**	1	**p** ***attR*** ** : 21 bp**	ND	5C3158057809	Intronic	Pcdh7				
	**6**	1	**p** ***attR*** **: No**	ND	6G1135251898	Intergenic		Gsg1	7	1700023A18Rik	51
	**7**	1	**p** ***attR*** **: 4 bp**	ND	7A17104238	Intergenic		AIE1	90	5730403M16Rik	10
	**9**	1	**p** ***attR*** **: 12 bp**	ND	9A13001529	Intronic	AC131780.5-201				
	**13**	1	**p** ***attR*** **: No**	ND	13D1102691720	Intergenic		AA414921	984	F630107B15	1.5
	**17**	1	**p** ***attR*** **: No**	ND	17A3.323698186	Intergenic		EG622645	305	4.1B	53
	**X**	1	**p** ***attR*** **: 10 bp**	ND	XA7.375331158	Intronic	Cf-8				
**Human**											
**Groupe I**	**1**	2	**p** ***attR*** **: No** **p** ***attL*** **: 20 bp**	**13 bp**	1q32.1205953621/634	Intergenic		SLC26A9	15	RAB7	28
	**9**	1	**p** ***attR*** **: 9 bp** **p** ***attL*** **: ND**	ND	9p21.133615936	Intergenic		bA255A11.3	43	TCRBV20S2	1.5
	**17**	2	**p** ***attR*** **: No** **p** ***attL*** **: 12 bp**	**Inversion of 4,8 kb**	17q11.226164583/59669	Intronic	HSD24				
	**17**	1	**p** ***attR*** **: No** **p** ***attR*** **: ND**	ND	17q21.3245406611	Intronic	C17orf57				
	**17**	2	**p** ***attR*** **: 10 bp** **p** ***attL*** **: 34 bp**	**795 pb**	17q25.382068091/886	Intergenic		DUS1L	2559	FASN	11080

Twelve murine integration sites were isolated, three from group I with the two junctions (pa*ttL* and pa*ttR*) and nine from group III with only the pa*ttR* junction (Table 2). For two of the three group I clones both flanking regions were sequenced but the isolated junctions were too short to allow identification of the integration locus. Surprisingly, these two clones of group I present abnormal p*attL* and p*att*R junctions where several bases were missing, probably due to a deletion mechanism. Similarly, the sequence analysis of group III clones shows that recombination between the *attB* site and p*attP* site is not as precise as expected. Indeed, 6 out of 9 clones have missing bases in the p*attR* junctions. However, because we isolated only one flanking region for clones of group III, we cannot conclude about deletion events, as the missing bases could result from a gap of the a*ttB* core region involved in recombination.

**Figure 7:**
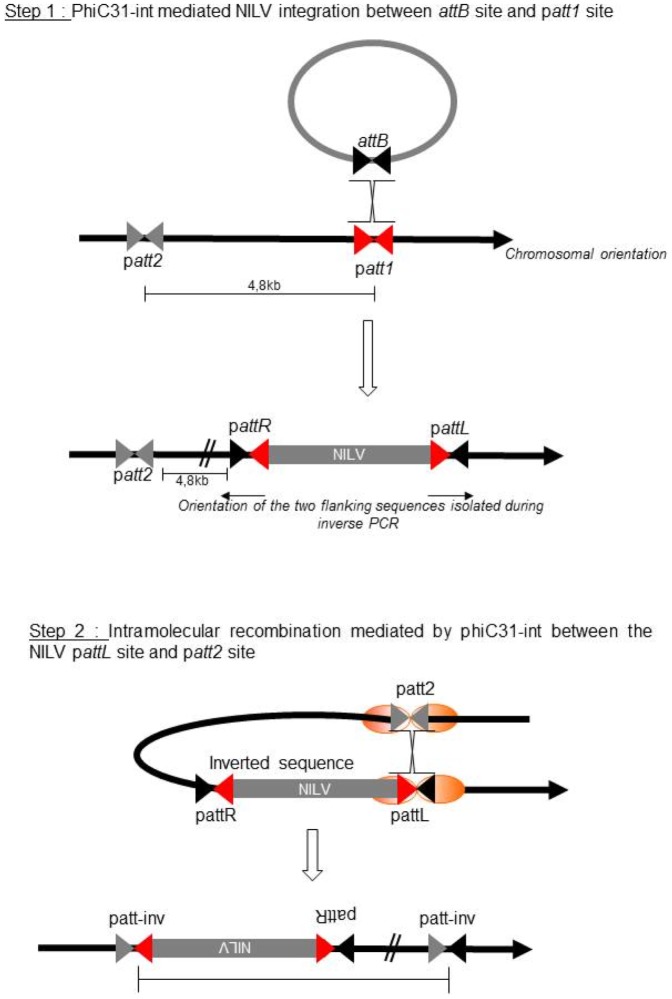
Hypothetical model to explain the inversion of 4.8 Step 1: Integration of a NILV mediated by phiC31-int into a p*attP* site. Step2: Recombination mediated by phiC31-int between the p*attL* generated during step 1 and another p*att* site located at 4 kb.

Five human integration sites were isolated, all from group I. Both junctions were isolated for three sites and only the p*attR* junction for two sites (Table 2). As for the murine integration site analysis, missing bases in the recombined pseudo-sites were detected, including in clones for which both L and R junction could be determined. This further confirms that missing bases indeed reveal a deletion mechanism, probably occurring during the phiC31-int mediated recombination between a natural *attB* site and a p*attP* site. Furthermore, the integration site of the three sites for which the two junctions were isolated could be localized exactly (Table 2). Interestingly, for these three sites chromosomal gaps were observed between the p*attR* site and the p*attL* site. The size gaps are 13 bp, 795 pb and 4.8 kbp. The two first gaps could result from a mechanism of deletion which could occur through NHEJ pathway. Nevertheless, these hypotheses cannot explain the gap of 4.8 kbp. Indeed, in this case, the two flanking regions isolated during iPCR are in the same chromosomal orientation, which is not the case when normal recombination occurs. We hypothesize that the observed aberrant recombination results from two successive recombination events involving two p*attP* sites located 4.8 kb from each other that led to an inversion of the 4.8 kbp sequence (figure 7). This mechanistic model was previously proposed to explain chromosomal rearrangements in mammalian cells resulting from aberrant recombinations mediated by phiC31-int [86].

In conclusion the hybrid lentivirus-phiC31-int-NLS vector that we developed allows targeted integration in p*attP* sites in murine and human genomes but seem to induce frequent deletions of base pairs in the *attB* site present into the vector or into the endogenous p*attP* site. Moreover, it may induce chromosomal deletions and translocations.

## Conclusions

Our work establishes the ability of the hybrid lentivirus-phiC31-int-NLS to integrate a NILV substrate into a*ttP* or pa*ttP* sites in murine and human cell lines. Although the number of integration sites isolated in this study does not allow to determine preferential genome recognition sites for integration mediated by phiC31-int vectorized in NILV, a human p*attP* site already described could be isolated [66]. Most importantly, we demonstrated that the use of hybrid lentiviral phiC31-int-NLS vector can induce DNA damages, probably due to the activity of the recombinase. Indeed, other reports already described similar results using non-viral transfection of PhiC31-int. Anja Ehrhardt et al have shown that 15% of transgenes integrated by phiC31-int were flanked by chromosomal DNA sequence from different chromosomes [86] and Ji Liu et al have shown that phiC31-int induces DNA damages and chromosomal rearrangements in primary and adult human fibroblasts [87], [88]. In addition, Ehrard et al. have shown that phiC31-int is competent to integrate linear DNA fragments. They did not characterize the mechanisms and the consequences of this type of event are unknown, but one may hypothesize that such event would induce chromosome break. Considering that the cycle of NILVs involves linear intermediates, it would be of particular interest to further investigate the ability of phiC31-int to integrate these linear forms and the consequences of such events. Taken together, these observations limit the use of the hybrid lentiviral phiC31-int-NLS vector for clinical applications, it remains useful in transgenesis contexts where non aberrant recombination events can be selected. Alternatively, the hybrid lentiviral system may be used to vectorize other recombinases or genome editing tools with higher safety features. Indeed other serine recombinases have been shown to function in human cells [89]–[91] and could be valuable candidates for vectorization in NILVs. Moreover, the modification of recombinases and other genome editing tools by directed evolution techniques [92], [93] could be used to render them hyper-specific and hyper-efficient in order to improve their safety features. For instance, directed evolution has proven efficient to modify the efficiency and/or specify of a variety of molecular tools, including Sleeping Beauty [94] Cre recombinase [95], FLP recombinase [96], ZFNs [97] or phiC31-int [98].

## Methods

### Plasmids

The encapsidation plasmid expressing a functional integrase (p8.91 IN_WT_) has been described previously [77]. The encapsidation plasmid expressing a deficient integrase (p8.91 IN_D64V_) was derived from the plasmid p8.91 IN_WT_ and the plasmid pCMVΔR(int-)8.2 previously described [74] and kindly provided by D.B. Kohn (UCLA, Los Angeles (CA), USA). This plasmid contains a point mutation in the coding region of the integrase catalytic domain, creating a D64V change in the amino acid sequence. The plasmid p8.91 IN_D64V_ was generated by replacing the BclI-AflII fragment of p8.91 IN_WT_ by the corresponding fragment (containing the substitution) from pCMVΔR(int-)8.2.

The envelope expression plasmid pMDG(VSV) was used to express the VSV-G from the human CMV immediate early promoter [74].

The vector plasmid pTrip-CMV-phic31-int-WPRE was derived from the plasmid pTrip-CMV-GFP-WPRE previously described [77] and the plasmid pCMV-phic31-int previously described [41] and kindly provided by M.P. Calos (Stanford University, Stanford (CA), USA). The plasmid pTrip-CMV-phic31-int-WPRE was generated by replacing the BamHI-SnabI fragment (CMV-GFP) of the pTrip-CMV-GFP-WPRE by the CMV-phic31-int fragment from the plasmid pCMV-phic31-int. The vector plasmid pTrip-CMV-phic31-int-NLS-WPRE was derived from the plasmid pTrip-CMV-phic31-int-WPRE and the plasmid pCMV-phic31-int. The C-terminal region of phic31-int was amplified with a primer containing the SV40 NLS sequence (5′-CCCGTTGGCAGGAAGCACTTCCGG-3′/5′- ATTCGCGGATCCGCTAAACCTTCCTCTTCTTCTTAGGCGCCGCTACGTCTTCCGTGCCGTCC-3′) from the plasmid pCMV-phic31-int. This PCR product was subcloned into the plasmid pCMV-phic31-int in place of the C-terminal region of phic31-int by using Eco47III and BamHI restriction enzymes to generate the plasmid pCMV-phic31-int-NLS. The plasmid pTrip-CMV-phic31-int-NLS-WPRE was finally generated by replacing the SpeI-BamHI fragment (GFP) of the pTrip-CMV-phic31-int-WPRE by the corresponding fragment (phic31-int-NLS) from pCMV-phic31-int-NLS. The vector plasmid pTrip-*attB*-CMV-Neo-WPRE was derived from the plasmid pTrip-CMV-Neo-WPRE previously described [77] and the plasmid p*attB* previously described [41] and kindly provided by MP Calos. The plasmid pTrip-a*ttB*-CMV-Neo-WPRE was generated by inserting the SalI fragment of the pa*ttB* plasmid (a*ttB* sequence) into the linearized plasmid pTrip-CMV-Neo-WPRE.

The plasmid pTrip-CMV-a*ttP*-DsRed2 was derived from the plasmid pTrip-CMV-DsRed2 kindly provided by P. Ravassard. Hybridization of 2 single strands DNA fragments (5′- CCCCAACTGGGGTAACCTTTGAGTTCTCTCAGTTGGGGG-3′/5′-CCCCCAACTGAGAGAACTCAAAGGTTACCCCAGTTGGGG-3′) generated a double stranded DNA fragment corresponding to the a*ttP* sequence flanked by BamHI cohesive ends. This fragment was inserted into the linearized plasmid pTrip-CMV-a*ttP*-DsRed2 to generate the plasmid pTrip-CMV-a*ttP*-DsRed2.

### Lentiviral production

Lentiviral vectors were generated by the transient transfection of 293T cells by using the calcium phosphate precipitation method previously described [77]. Briefly, cells were cotransfected with the vector plasmid (pTrip-CMV-phic31-int-WPRE, pTrip-CMV-phic31-int-NLS-WPRE, pTrip-a*ttB*-CMV-Neo-WPRE or pTrip-CMV-a*ttP*-DSred2-WPRE), the transcomplementation plasmid (p8.91 IN_WT_ for integrative vectors or p8.91 IN_64_ for non-integrative vectors), and the plasmid encoding the vesicular stomatitis virus envelope glycoprotein (pMD-G). Supernatant was collected 48 hours after transfection, treated with DNaseI (Roche) and filtered (0.45 µm). Viral particles were then concentrated by ultracentrifugation (90 min, 22,000 rpm, rotor SW28) and resuspended in 0.1M PBS. The HIV p24 Gag antigen was quantified for each stock by ELISA (HIV-1 P24 antigen assay; Beckman Coulter, Fullerton, CA) according to manufacturer's instructions.

### Cell culture

Human epithelial HeLa, Hi16, HeLa-DsRED2 and 293T cells and murine NIH 3T3 cells were grown in Dulbecco's modified medium (Invitrogen) supplemented with antibiotics (100 U/mL penicillin and 100 mg/mL streptomycin) and 10% heat inactivated fetal calf serum (Eurobio). The cells were plated and cultured in a humidified incubator at 37°C in a 5% CO2 and 90% air atmosphere.

### Genomic DNA extraction

Genomic DNA extractions were performed with a lysis buffer composed of TrisHCl 10 mM (pH 7.5), EDTA 10 mM, SDS 0.6%, RNase A (Qiagen) 100 µg/mL and proteinase K (Eurobio) 100 µg/mL. The lysates were purified by phenol/chloroform and precipitated using sodium acetate and ethanol.

### Generation of a reporter cell line HeLa-DsRED2

The HeLa-DsRED2 cell line containing an a*ttP* site and expressing DsRED2 fluorescent protein was generated using the lentiviral vector CMV-a*ttP*-DsRED2. HeLa cells were transduced with LV CMV-a*ttP*-DsRED2 (unconcentrated supernatant). Cells were grown for 3 days and seeded at clonal density in 96-well plates (0.3 cell per well) to generate single cell derived colonies. Clones were analyzed for DsRED2 expression by flow cytometry and PCR amplification of the vector genome.

### Transduction

Cell suspensions were incubated for 3 hours with required vectors in medium supplemented with 1 µM DEAE-dextran. After 3 hours of incubation, cells were seed at desired density in fresh medium and grown for the purpose of the experiment.

Hi16 cells (2.10^6^ cells/mL) were transduced with NILV-phic31-int (300 ng of p24) and NILV-Neo (100 ng of p24). Cells were seeded in 10 cm plates and grown in medium supplemented with 1 mg/mL of G418 for 12 days. Cells were then seeded in 96 well plates at low density (0.3 cell per well) to generate single cell derived colonies.

HeLa DsRED2 cells (2.10^6^ cells/mL) were transduced with NILV CMV-phic31-int (50, 150, 300 ng of p24) and NILV CMV-Neo (100 ng of p24). Cells were seeded in 10 cm plates and grown in medium supplemented with 1 mg/mL of G418 (renewed every 3 days) for 12 days before extraction and analysis of genomic DNA.

Hela and NIH-3T3 cells (5.10^5^ cells/mL) were transduced with NILV CMV-phic31-int (300 ng of p24) and NILV CMV-Neo (100 ng of p24). Cells were seeded in 10 cm plates and grown in medium supplemented with 1 mg/mL of G418 for 12 days. Cells were then seeded in 96 well plates at low density (0.3 cell per well) to generate single cell derived colonies.

### Evaluation of Recombination Frequency

HeLa were directly transduced in suspension (8.10^4^ cells/mL) with NILV CMV-phic31-int (3, 6, 18 and 36 ng of p24) and NILV CMV-Neo (12 ng of p24) during 3 hours in 150 µL of medium supplemented with 1 µM DEAE-dextran. Cells were then seeded in 6-wells plates with 2 mL of fresh medium. The day after, medium was removed and replaced with fresh medium supplemented with 1 mg/mL of G418. The medium was replaced every 3 days. Cells were grown 12 days, until clones developed and were then fixed with PFA 4% and stained with trypan blue. Clones on each well were counted. We transduced cells in three replicate tubes for each condition, and results are expressed as the mean of three measurements.

### PCR reactions

#### RT-PCR

To analyze phic31-int expression by RT-PCR, total RNAs were extracted from HeLa cells using the RNeasy minikit (Qiagen), according to the manufacturer's instructions. Then, RNAs were reverse transcribed using the Superscript First Strand Synthesis kit (Invitrogen), according to the manufacturer's instructions. Phic31-int cDNA was amplified using the primers 5′-GCGAAGATTCTCGACACG-3′ and 5′-TCGCAGTACAGCTTGTCC-3′ at the concentration of 10 µM.

#### PCR on genomic DNA

PCR performed on genomic DNA used 500 ng of DNA, 1.5 mM of MgCl2 and 10 µM of each primer. The primers were as follows: amplification of a*ttB* region: 5′-CAATTTGCTGAGGGCTATTGAG-3′ and 5′- CTGTCCCTGTAATAAACCCG-3′; amplification of the LTRs region: 5′-CTCAATAAAGCTTGCCTTGAGTGC-3′ and 5′-TCAGATCTGGTCTAACCAGAGAGACC-3′; amplification of the DsRed2 region: 5′-AGGCCAGACAATTATTGTCTGG-3′ and 5′-ATGGTCTTCTTCTGCATCACG-3′; amplification of DsRED2 (nested primers) 5′- AAGAATCCTGGCTGTGGAAAG-3′ and 5′-AACTCGGTGATGACGTTCTCG-3′

#### Inverse PCR

Genomic DNA (10 µg) was primarily submitted to enzymatic restriction. The enzymatic cocktail used was: XbaI (100 U), StuI (100 U) BsrGI (30 U) and BstXI (30 U). After 16 hours incubation, the enzymes were heat inactived at 65°C for 30 minutes. Cohesive ends were filled in with Klenow (15 U) and dNTPs (2 mM) at 25°C for 20 minutes. Klenow was inactived with 1 mM EDTA. The products of digestion were purified by phenol/chloroform and precipitated using sodium acetate and ethanol. The ligation of the digestion products was performed by ligase (1.000 U, NEB) within ligation buffer supplemented with ATP (1 mM). The products of ligation were purified by phenol/chloroform and precipitated using sodium acetate and ethanol. PCR was performed with 100 ng of DNA with primers allowing the amplification of the a*ttB* region.

Adapted inverse PCR was performed using the same protocol with the addition of BmtI (30 U) in the enzymatic restriction cocktail.

The products of inverse PCR were visualized on 0.8% agarose gel with ethidium bromide staining. These products were extracted and purified using the Wizard SV Gel and PCR Clean-up System (Promega) according to the manufacturer's instructions, then cloned in a plasmid using pGEM-T Easy Vector System I according to the manufacturer's instructions. Next, inverse PCR products were sequenced using T7 and/or Sp6 primers.

### Sequence analysis

All sequencing was performed by Eurofins genomics. Sequences were aligned with vector and genomic DNA, and recombination junctions were identified by sequence matching to a*ttB*. Human and murine blasts were performed using the *NCBI* and *ensembl genome* databases. The chromosomal localization of pseudosites attP has been performed using the Genebank GRCm38.p2 C57BL/6J assembly in mouse and the Primary Assembly GRCh38 for Human.
